# Digoxin attenuates LPS-induced acute lung injury in mice via NF-κB and HIF-1α inhibition

**DOI:** 10.17305/bb.2026.13786

**Published:** 2026-02-16

**Authors:** Abdulaziz M Alzahrani, Mai A Alim A Sattar Ahmad, Wafaa S Ramadan, Huda M Alkreathy

**Affiliations:** 1 Department of Clinical Pharmacology, Faculty of Medicine, King Abdulaziz University, Jeddah, Saudi Arabia; 2 Department of Pharmaceutical Care, King Abdulaziz Hospital, Makkah, Saudi Arabia; 3 Department of Anatomy, Faculty of Medicine, King Abdulaziz University, Jeddah, Saudi Arabia

**Keywords:** Digoxin, lipopolysaccharides, acute lung injury, NF-kappa B, hypoxia-inducible factor 1, alpha subunit

## Abstract

Acute lung injury (ALI), which can progress to the highly lethal acute respiratory distress syndrome (ARDS), remains a condition with limited pharmacological interventions, highlighting the urgent need for mechanism-informed repurposing strategies. Given the reported inhibitory effects of digoxin on nuclear factor kappa B (NF-κB)-mediated inflammation, we assessed its prophylactic and therapeutic efficacy in a murine model of lipopolysaccharide (LPS)-induced ALI. Sixty-six adult male BALB/c mice were divided into control and LPS-challenged groups. The LPS group (5 mg/kg, intratracheal) was assigned to either prophylactic regimens (daily saline, dexamethasone 5 mg/kg, or digoxin 1 mg/kg, administered intraperitoneally for 5 days prior to LPS) or therapeutic regimens (same interventions initiated 12 h post-LPS for 3 days). At the study endpoint, pulmonary edema (wet-to-dry ratio) and lung injury pathways were analyzed in lung homogenates, including tumor necrosis factor-alpha (TNF-α), IL-6, myeloperoxidase (MPO), malondialdehyde (MDA), NF-κB (p65) DNA-binding activity, C-X-C motif chemokine ligand 2/macrophage inflammatory protein-2 (CXCL2/MIP-2), and hypoxia-inducible factor-1 alpha (HIF-1α), alongside histopathological evaluations. Digoxin significantly reduced pulmonary edema in the therapeutic group (*P* ═ 0.02) and decreased TNF-α levels (*P* ═ 0.014), while robustly suppressing IL-6 and MDA in both prophylactic and therapeutic settings (each *P <* 0.001). Moreover, digoxin lowered MPO levels prophylactically (*P* ═ 0.007) and therapeutically (*P* ═ 0.014). It also strongly inhibited NF-κB activation, reducing CXCL2/MIP-2, while decreasing HIF-1α in both regimens (each *P <* 0.001). Histological analysis corroborated these findings, revealing improved alveolar architecture and reduced inflammatory injury. In conclusion, digoxin exhibits potent immunomodulatory activity in experimental ALI, warranting further translational research focused on dose optimization and safety profiling.

## Introduction

Acute respiratory distress syndrome (ARDS) poses a significant challenge in critical care medicine, representing a substantial health burden. The early inflammatory phase of ARDS, previously referred to as acute lung injury (ALI) [1], remains the established experimental model. The complex nature of ARDS, particularly emphasized during the COVID-19 pandemic, not only jeopardizes patient survival but also strains medical expertise and healthcare infrastructure [2]. Addressing these challenges requires an integrated response that includes enhanced clinical vigilance, optimized treatment protocols, and the development of innovative therapeutic strategies to address the limitations of current management approaches.

The LUNG-SAFE study, a pivotal multicenter observational cohort study, found that ARDS accounts for approximately one-tenth of all intensive care unit (ICU) admissions and is frequently underrecognized and undertreated. Hospital mortality rates correlate with ARDS severity, with approximately 35% for mild, 40% for moderate, and over 46% for severe cases [3]. Additionally, ARDS survivors often experience long-term morbidities, including physical impairments, pulmonary dysfunction, cognitive deficits, and psychological disorders, all of which detract from quality of life and increase healthcare utilization [4, 5]. Moreover, the financial burden of ARDS impacts 67% of patients, with hospitalization costs potentially exceeding $100,000 [6, 7]. These factors underscore the necessity for ongoing research into effective ARDS interventions.

Clinically, ARDS comprises a spectrum of severe respiratory manifestations characterized by diffuse lung inflammation and impaired gas exchange. These conditions are most commonly precipitated by pneumonia, which is the leading cause, as well as by sepsis, aspiration, or major trauma [8]. Inflammation is a central feature, leading to an influx of neutrophils into the airways and subsequent disruption of the capillary-alveolar membrane, resulting in hypoxemia and pulmonary edema [9, 10]. Notably, the regulatory mechanism of nuclear factor kappa B (NF-κB) plays a significant role in ARDS pathogenesis. NF-κB signaling has been shown to orchestrate proinflammatory gene expression, regulate inflammasome activation, and control the production of key adhesion molecules, thus facilitating leukocyte recruitment and amplifying inflammatory cascades [11–13]. Furthermore, dysregulated NF-κB activation in ARDS drives cytokine storms and neutrophilic lung infiltration, with the duration and magnitude of activity correlating with injury severity [14, 15]. The cytokine storm associated with COVID-19 is largely mediated by NF-κB signaling, suggesting that inhibition of this pathway may represent a promising therapeutic strategy [16].

A screening study of 2,800 bioactive compounds identified cardiac glycosides, including digoxin, as among the few drugs with potent NF-κB inhibitory properties [17]. Importantly, digoxin exhibits significant anti-inflammatory effects attributed to its modulation of the NF-κB regulatory pathway [18]. Additionally, digoxin suppresses NF-κB signaling activity triggered by tumor necrosis factor-alpha (TNF-α) and interleukin-1 beta (IL-1β), while also demonstrating promising antiviral activity [17–19]. Digoxin’s therapeutic potential extends beyond cardiac applications; a randomized double-blind trial found that adjunctive digoxin improved outcomes and reduced inflammatory markers in rheumatoid arthritis patients through NF-κB inhibition of cell differentiation and cytokine production [20]. Furthermore, animal studies have established digoxin’s efficacy in treating both alcoholic and nonalcoholic steatohepatitis [21]. Research has also shown that digoxin suppresses the expression of hypoxia-inducible factor 1-alpha (HIF-1α), a transcription factor critically involved in modulating inflammatory responses and tissue damage in ARDS [22, 23].

While literature has discussed whether digoxin merits consideration over other therapeutic options for COVID-19 patients with atrial arrhythmias, this notion has faced substantial criticism due to its uncertain efficacy, recognized toxicity risks, and the lack of definitive clinical evidence [24, 25]. Building on these encouraging findings, the present investigation aims to evaluate digoxin’s prophylactic and therapeutic effects in a mouse model of ALI, thereby providing preclinical insights relevant to the inflammatory aspects of ARDS.

## Materials and methods

### Animals and experimental design

**Figure 1.**
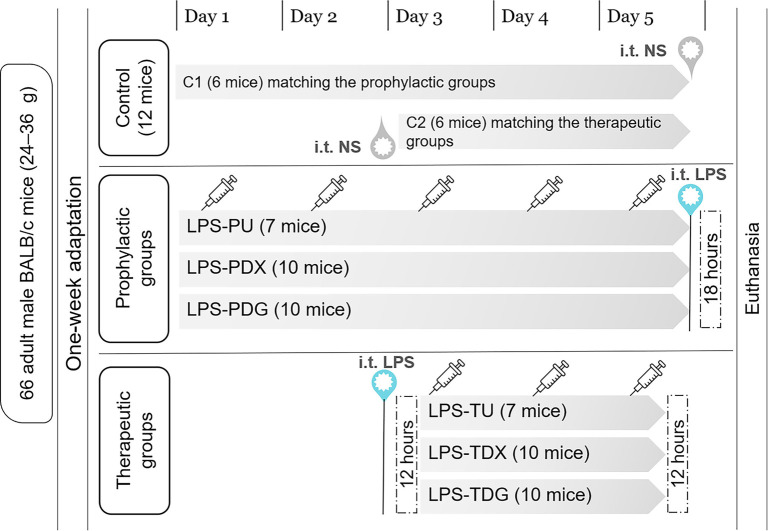
**Timeline of experimental design for mouse studies.** Control mice were evenly divided and received i.t. administration of a weight-based volume of NS. The LPS groups included all experimental groups receiving i.t. instillation of LPS (5 mg/kg) to induce ALI in both prophylactic and therapeutic settings. The prophylactic groups consisted of: LPS-PU (untreated), LPS-PDX (i.p. dexamethasone 5 mg/kg), and LPS-PDG (i.p. digoxin 1 mg/kg). The therapeutic groups included: LPS-TU (untreated), LPS-TDX (i.p. dexamethasone 5 mg/kg), and LPS-TDG (i.p. digoxin 1 mg/kg). Abbreviations: ALI: Acute lung injury; i.p.: Intraperitoneal; i.t.: Intratracheal; LPS: Lipopolysaccharide; LPS-PDG: Lipopolysaccharide–prophylactic digoxin; LPS-PDX: Lipopolysaccharide–prophylactic dexamethasone; LPS-PU: Lipopolysaccharide–prophylactic untreated; LPS-TDG: Lipopolysaccharide–therapeutic digoxin; LPS-TDX: Lipopolysaccharide–therapeutic dexamethasone; LPS-TU: Lipopolysaccharide–therapeutic untreated; NS: Normal saline.

Sixty-six adult male BALB/c mice (24–36 g) were obtained from the animal facility at King Saud University (Riyadh, Saudi Arabia). Throughout the experimental period, the animals were housed under standardized conditions, with ambient temperatures maintained between 29 ^∘^C and 30 ^∘^C. A consistent circadian rhythm was established using a 12-hour light/dark cycle, and dietary requirements were met by providing ad libitum access to feed and tap water. Following a one-week adaptation phase, the animals were randomly allocated into seven groups to investigate the effects of prophylactic and therapeutic interventions on ALI induced by lipopolysaccharide (LPS) (Figure 1). The LPS-induced ALI model was selected because it replicates several key features of human ARDS and is widely accepted for investigating the pathophysiology and potential therapeutics for lung injury [26, 27]. The experimental design included a **control** group (**C**, *n* ═ 12), which was evenly divided (C1 and C2) to match either the prophylactic or therapeutic groups. Additionally, six LPS-challenged groups were established and further subdivided based on the timing and type of intervention administered. The interventions included once-daily administration of either normal saline (0.9% NaCl), dexamethasone (EPICO, Tenth of Ramadan City, Egypt), or digoxin (ANFARM Hellas S.A., Schimatari, Viotia, Greece), as detailed below:

### Prophylactic groups received the respective interventions for five days prior to LPS administration:

- **LPS-prophylactic untreated** (**LPS-PU**, *n* ═ 7): received an equivalent volume of intraperitoneal (i.p.) normal saline prior to LPS challenge.
- **LPS-prophylactic dexamethasone** (**LPS-PDX**, *n* ═ 10): pretreated with i.p. dexamethasone (5 mg/kg) [28] before LPS administration.
- **LPS-prophylactic digoxin** (**LPS-PDG**, *n* ═ 10): administered digoxin (1 mg/kg, i.p.) [29, 30] before LPS exposure.

### Therapeutic groups commenced the respective intervention 12 h post-LPS exposure:

- **LPS-therapeutic untreated** (**LPS-TU**, *n* ═ 7): received an equivalent volume of i.p. normal saline for three days post-LPS administration.
- **LPS-therapeutic dexamethasone** (**LPS-TDX**, *n* ═ 10): treated with i.p. dexamethasone (5 mg/kg) for three days following LPS challenge.
- **LPS-therapeutic digoxin** (**LPS-TDG**, *n* ═ 10): given i.p. digoxin (1 mg/kg) for three days post-LPS induction.

All mice were anesthetized and humanely euthanized by cervical dislocation at the end of the experiment. In the prophylactic experiment (C1, LPS-PU, LPS-PDX, and LPS-PDG), euthanasia was performed 18 h after model induction, a time point that may optimally capture elevated inflammatory biomarkers, peak neutrophil migration into alveolar spaces, pronounced histopathological manifestations [31], and pulmonary edema development. For the therapeutic portion (C2, LPS-TU, LPS-TDX, and LPS-TDG), animals were euthanized 12 h following the final administration of the assigned intervention, allowing sufficient time for drug accumulation and potential effects while preceding the probable initiation of the injury resolution phase [32].

### Open-drop anesthesia procedure

Anesthesia was utilized during the induction of the ALI model and at the conclusion of the experiment to ensure humane euthanasia. Mice were anesthetized with approximately 8% sevoflurane (Haluran, Tabuk Pharmaceuticals Manufacturing Co., Riyadh, Saudi Arabia) via the open-drop method. Sevoflurane was chosen for its low airway irritation potential [33], minimizing alterations to physiological responses in the experimental model. Specifically, 1 mL of liquid sevoflurane was placed on a cotton pad beneath a mesh floor in a 2.2-liter glass bell jar, preventing direct contact with the mice. The anesthetic procedure was conducted at a room temperature of 20^∘^C and an estimated atmospheric pressure of around 760 mmHg. Concentration and dosage were determined based on the methodology of Risling et al. [34] and established guidelines from *Anesthesia and Analgesia in Laboratory Animals* (3rd edition) [35], which outlined the approximate volumes needed to achieve specific concentrations in similar enclosed conditions. The chamber was ventilated and sterilized between animals, with a freshly prepared sevoflurane-soaked cotton pad placed for each mouse. All procedures were performed under a chemical fume hood to ensure operator safety and adequate ventilation. Mice were continuously monitored until deep anesthesia was confirmed by a reduced respiratory rate and the loss of withdrawal reflexes (tail pinch and pedal reflex).

### Induction of ALI model

To induce ALI, an established murine model using LPS from *Escherichia coli* O55:B5 (HY-D1056; MCE, NJ, USA) was employed. The procedure involved intratracheal (i.t.) administration of LPS following a weight-based dosing regimen. A concentrated LPS solution (5 µg/µL) was prepared and delivered at a weight-adjusted dosage of 5 mg/kg (i.e., 25 µL i.t. volume for a 25 g mouse). Prior to the procedure, mice were anesthetized as described above to facilitate intratracheal administration. LPS instillation was performed using a non-invasive technique to access the trachea [36]. Control animals received a corresponding weight-based volume of normal saline (vehicle) via the same route, serving as a comparative baseline for the study.

### Body weights and lung wet-to-dry weight ratio

Animal body weights (g) were measured at the beginning of each experiment and prior to sacrifice using a sensitive electronic balance. The lung wet-to-dry weight ratio was determined using a gravimetric method to quantify pulmonary edema. Following euthanasia, at least five right lungs per group were harvested and immediately weighed to determine their fluid-inclusive mass. After the initial weighing, samples were dried for 72 h in a precision oven maintained at 70 ^∘^C. The dried samples were subsequently weighed again. The wet-to-dry weight ratio, calculated as the ratio between the initial and dehydrated masses, provided a quantitative measure of lung water content.

### Pulmonary tissue homogenization and total protein quantification

The left and right lungs not allocated for other analyses (see Supplementary Table S1) were carefully excised, thoroughly rinsed with ice-cold isotonic saline, and cryopreserved at -80^∘^C until further processing. Prior to homogenization, frozen tissues were allowed to thaw gradually on ice. Using a sterile scalpel, the lung tissue was finely minced and transferred to 200 µL of ice-cold homogenization buffer (1:9 w/v tissue-to-buffer ratio) containing 0.1 M potassium phosphate (pH 7.5), 10% glycerol, 10 mM K2EDTA, 0.1 M dithiothreitol (DTT), and 0.39 mM phenylmethylsulfonyl fluoride (PMSF). The tissue suspension was homogenized using an Ultra-Turrax T 25 (IKA, Staufen, Germany) homogenizer to ensure thorough disruption of cellular structures and uniform distribution of lung components in the buffer. After homogenization, the mixture underwent ultrasonication and centrifugation to obtain the supernatant. Total protein concentration in the supernatant was measured using the BCA protein assay kit (MyBioSource, Inc., San Diego, CA, USA; Cat. No. MBS355529) with absorbance recorded at 562 nm. These values were utilized to normalize all subsequent biochemical analysis results to units of analyte per mg protein, thereby enhancing data precision and statistical robustness across samples. Lung tissue designated for nuclear NF-κB activity assay was processed using a nuclear extraction kit (Abnova Corporation, Taipei, Taiwan; Cat. No. KA1346), with the minced tissue homogenized in the kit-specific nuclear extraction hypotonic buffer and processed with a Dounce homogenizer (Fisher Scientific, Waltham, MA, USA), strictly adhering to the manufacturer’s instructions.

### Measurement of proinflammatory cytokines (TNF-α and IL-6)

Inflammatory mediators were quantified using enzyme-linked immunosorbent assay (ELISA) kits specific for murine TNF-α (My BioSource, Inc., San Diego, CA, USA; Cat. No. MBS2500421) and interleukin-6 (IL-6) (My BioSource, Inc., San Diego, CA, USA; Cat. No. MBS355431). The assays were conducted on lung tissue homogenates in strict accordance with the manufacturer’s instructions.

### Evaluation of myeloperoxidase (MPO) enzyme level and oxidative stress

Myeloperoxidase (MPO), a valuable biomarker for assessing lung injury and neutrophil accumulation in lung tissue, was quantified in lung homogenates using an ELISA kit (My BioSource, Inc., San Diego, CA, USA; Cat. No. MBS700747), following the supplier’s instructions. MPO levels may also indicate oxidative stress, alongside malondialdehyde (MDA), a well-established marker in this context [37, 38]. The concurrent evaluation of MDA and MPO provides a more comprehensive assessment of oxidative stress, enhancing the reliability of findings. MDA concentrations were quantified using a specific ELISA kit (My BioSource, Inc., San Diego, CA, USA; Cat. No. MBS741034), adhering strictly to the manufacturer’s guidelines.

### Assessment of NF-κB activation, CXCL2/macrophage inflammatory protein-2 (CXCL2/MIP-2) protein expression and regulatory influence on HIF-1α

NF-κB activation was evaluated by measuring its DNA binding activity using the NF-κB (p65) transcription factor assay kit (Abnova Corporation, Taipei, Taiwan; Cat. No. KA1341) on nuclear extracts. Additionally, the protein level of CXCL2/MIP-2, a principal downstream effector of NF-κB, was quantified in lung homogenates using the mouse CXCL2 ELISA kit (My BioSource, Inc., San Diego, CA, USA; Cat. No. MBS824972). Furthermore, the modulation of HIF-1α was investigated by determining HIF-1α protein levels with the mouse hypoxia-inducible factor 1 alpha (HIF1A) ELISA kit (My BioSource, Inc., San Diego, CA, USA; Cat. No. MBS9309033).

### Histopathological examination and scoring of lung injury

Left lungs from two mice in the control group, two mice in each LPS-untreated group (LPS-PU and LPS-TU), and four mice from each of the remaining groups were excised for histopathological assessment and scoring. The lungs were rinsed with phosphate-buffered saline (PBS) and fixed in 10% neutral-buffered formalin for 48 h. Fixed tissues were processed, embedded in paraffin, and sectioned at a thickness of 4 µm. Sections were stained with hematoxylin and eosin (H&E) and examined under a light microscope to assess histopathological changes in the lung tissue. Moreover, lung injury was evaluated unblinded using a semi-quantitative histopathological scoring system adapted from Franks et al. [39]. The following features relevant to the established model were assessed: airspace edema, thickening of alveolar septa, type II pneumocyte hyperplasia, hemorrhage, capillary congestion, prominent hyaline membrane formation, and accumulation of inflammatory cells. Each feature was recorded as present or absent (–). When present, the extent of parenchymal involvement was graded on a scale from 1–4, corresponding to mild (1, 1%–24% involvement), moderate (2, 25%–49%), marked (3, 50%–74%), and severe (4, 75%–100%). Slides prepared from each animal’s tissue block were divided into four quadrants for examination. For each animal, the total histopathological score was established by aggregating all individual feature scores, with absent features (–) assigned a value of zero.

### Statistical analyses

All statistical analyses were conducted using Statistical Package for the Social Sciences (SPSS) software version 25 (IBM SPSS, IBM Corp., Armonk, NY, USA). Data were processed and presented primarily as mean values with standard error of the mean (SEM). Unpaired two-sample *t*-tests were employed to compare all measured parameters between control groups C1 and C2 to identify any significant differences. For comparative purposes, wet-to-dry lung ratios and biomarker results were presented collectively; however, the prophylactic and therapeutic groups were analyzed separately relative to the control group. Data residuals were assessed for normality using Shapiro–Wilk tests. For normally distributed data (body weight change, TNF-α, IL-6, MDA, NF-κB activity, CXCL2/MIP-2, and HIF-1α), a one-way Welch ANOVA was conducted, followed by Games–Howell post hoc tests. For non-normally distributed data (lung wet-to-dry ratio and MPO), nonparametric Kruskal–Wallis tests were performed, followed by Dunn’s multiple-comparison post hoc tests with Bonferroni correction. To address multiplicity across the lung injury endpoints, Benjamini-Hochberg false discovery rate (BH-FDR) correction [40] was applied separately to *P* values from each group comparison. A significance level of α < .05 was established for all statistical tests.

**Table 1:** Changes in body weight among experimental mouse groups

	**Groups**	**Baseline body weight (g)**	**Terminal body weight (g)**	**Change in body weight (g)**
*Prophylactic experiment*	**Control (C1)**	32.8 ± 1.46	35.2 ± 1.34	2.4 ± 0.18
	**LPS-PU**	31.6 ± 1.48	32.3 ± 1.51	0.76 ± 0.13*
	**LPS-PDX**	32.3 ± 1.17	33.7 ± 1.03	1.34 ± 0.28*
	**LPS-PDG**	32.1 ± 1.13	32.4 ± 1.11	0.25 ± 0.11*
*Therapeutic experiment*	**Control (C2)**	30.6 ± 0.99	32.6 ± 0.87	2.01 ± 0.12
	**LPS-TU**	31.1 ± 1.16	31.1 ± 0.98	-- 0.06 ± 0.34*
	**LPS-TDX**	31.5 ± 0.95	32.3 ± 0.94	0.77 ± 0.34*
	**LPS-TDG**	31.7 ± 1.01	31.1 ± 1.04	-- 0.61 ± 0.27*

**Notes:** Data are presented as mean ± SEM. Weight measurements are expressed in grams (g). The control group consisted of 12 mice, evenly divided between the prophylactic (C1; *n* ═ 6) and therapeutic (C2; *n* ═ 6) conditions of the experiment. The LPS groups included all experimental groups that received i.t. instillation of LPS at a dosage of 5 mg/kg to induce ALI in both prophylactic and therapeutic groups. The prophylactic groups included: LPS-PU (untreated; *n* ═ 7), LPS-PDX (i.p. dexamethasone 5 mg/kg; *n* ═ 10), and LPS-PDG (i.p. digoxin 1 mg/kg; *n* ═ 10). The therapeutic groups included: LPS-TU (untreated; *n* ═ 7), LPS-TDX (i.p. dexamethasone 5 mg/kg; *n* ═ 10), and LPS-TDG (i.p. digoxin 1 mg/kg; n = 10). Statistical significance was determined using Welch’s ANOVA with Games-Howell post hoc tests, comparing each group to the corresponding control (C1 or C2). Prophylactic group comparisons (vs. C1) yielded the following results: LPS-PU (*P <* .001), LPS-PDX (*P ═* .03), LPS-PDG (*P <* .001). Therapeutic group comparisons (vs. C2) showed: LPS-TU (*P <* .001), LPS-TDX (*P ═* .006), LPS-TDG (*P <* .001). Abbreviations: ALI: Acute lung injury; ANOVA: Analysis of variance; i.p.: Intraperitoneal; i.t.: Intratracheal; LPS: Lipopolysaccharide; LPS-PDG: Lipopolysaccharide–prophylactic digoxin; LPS-PDX: Lipopolysaccharide–prophylactic dexamethasone; LPS-PU: Lipopolysaccharide–prophylactic untreated; LPS-TDG: Lipopolysaccharide–therapeutic digoxin; LPS-TDX: Lipopolysaccharide–therapeutic dexamethasone; LPS-TU: Lipopolysaccharide–therapeutic untreated; SEM: Standard error of the mean.

### Ethical statement

The animal experiments underwent comprehensive ethical assessment and received clearance from the Research Ethics Committee (REC No. PH-1445-34) at King Abdulaziz University (Jeddah, Saudi Arabia), and were conducted in accordance with the approved protocol. Additionally, the anesthesia and euthanasia protocols adhered to guidelines approved by the National Research Council (US) Committee on Recognition and Alleviation of Pain in Laboratory Animals [41], as well as the American Veterinary Medical Association (AVMA) Guidelines for the Euthanasia of Animals: 2020 Edition [42].

## Results

No mortality was observed among the mice throughout the experimental period. Except for body weight changes, groups C1 and C2 exhibited equivalent means and variances across all parameters tested, including the wet-to-dry lung weight ratio and biomarkers (TNF-α, IL-6, MPO, MDA, NF-κB, CXCL2/MIP-2, HIF-1α) (all *P* > .05). Consequently, data from control groups (C1 and C2) were pooled as a reference for subsequent comparisons with both prophylactic and therapeutic groups. Moreover, all nominally significant *P* values reported below survived BH-FDR adjustment (*q* < .05; Table S2).

### Effect of digoxin on body weights and lung wet-to-dry weight ratio

Initially, the experimental groups exhibited comparable body weights, with no statistically significant differences detected (Table 1). Conversely, at the end of the study period, Control group C1 demonstrated a significantly greater body weight gain than the prophylactic LPS-treated groups (LPS-PU, *P <* .001; *LPS-PDX, P* ═ .03; LPS-PDG, *P <* .001). Control group C2 also showed a marked increase compared to the therapeutic LPS-treated groups (LPS-TU, *P <* .001; LPS-TDX, *P* ═ .006; LPS-TDG, *P <* .001). Although no substantial differences were detected among the LPS groups, noteworthy trends in body weight changes emerged. In both the prophylactic and therapeutic groups, dexamethasone administration was associated with a marginally attenuated change in body weight. In contrast, mice receiving digoxin exhibited a slightly more pronounced reduction in weight gain; however, these differences did not reach statistical significance (*P* > .05).

**Figure 2.**
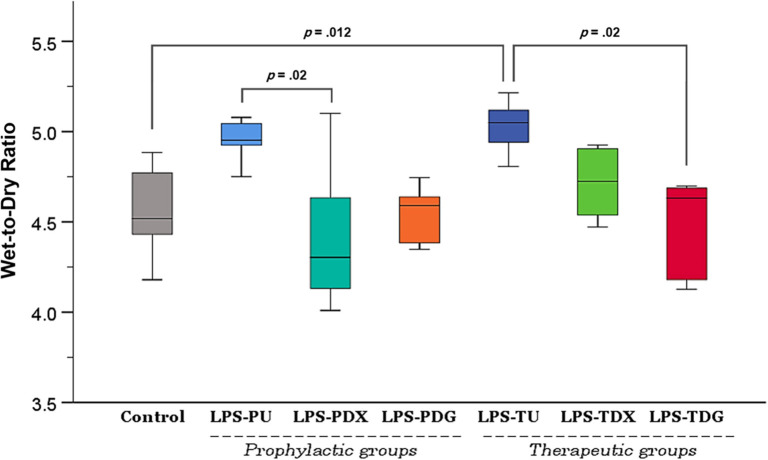
**Effect of digoxin on lung wet-to-dry ratio across different mouse groups.** Control mice (*n* ═ 10) were evenly combined from C1 and C2. The LPS groups include all mice that received intratracheal LPS (5 mg/kg) for the induction of ALI, further divided into prophylactic and therapeutic subgroups. Prophylactic subgroups: LPS-PU (untreated; *n* ═ 5), LPS-PDX (intraperitoneal dexamethasone 5 mg/kg; *n* ═ 6), and LPS-PDG (intraperitoneal digoxin 1 mg/kg; *n* ═ 6). Therapeutic subgroups: LPS-TU (untreated; *n* ═ 5), LPS-TDX (intraperitoneal dexamethasone 5 mg/kg; *n* ═ 6), and LPS-TDG (intraperitoneal digoxin 1 mg/kg; *n* ═ 6). Post-hoc Dunn-Bonferroni tests (Kruskal-Wallis) are indicated by brackets. Abbreviations: ALI: Acute lung injury; i.t.: Intratracheal; LPS: Lipopolysaccharide; LPS-PDG: Lipopolysaccharide–prophylactic digoxin; LPS-PDX: Lipopolysaccharide–prophylactic dexamethasone; LPS-PU: Lipopolysaccharide–prophylactic untreated; LPS-TDG: Lipopolysaccharide–therapeutic digoxin; LPS-TDX: Lipopolysaccharide–therapeutic dexamethasone; LPS-TU: Lipopolysaccharide–therapeutic untreated.

In the analysis of lung wet-to-dry weight ratio (Figure 2), the prophylactically untreated group (LPS-PU) showed an elevated yet statistically insignificant change (*P ═ * .12 vs control), indicating unremarkable pulmonary edema. Among the prophylactic intervention groups, only LPS-PDX group demonstrated a statistically significant reduction (*P ═* .02) in the wet-to-dry ratio compared to the LPS-PU group. Nevertheless, this result may be considered inconclusive due to the lack of a significant difference between the control and LPS-PU groups. In contrast, variations in this parameter were more pronounced in the therapeutic segment of the experiment. Specifically, the LPS-TU group exhibited a significant elevation (*P ═* .012) compared to baseline controls, reflecting pronounced pulmonary edema. Conversely, both dexamethasone (LPS-TDX) and digoxin (LPS-TDG) treatments demonstrated notable reductions in fluid accumulation compared to the LPS-TU mice. However, only the digoxin treatment achieved a statistically significant reduction (*P ═* .02).

### Effect of digoxin on proinflammatory cytokines (TNF-α and IL-6)

**Figure 3.**
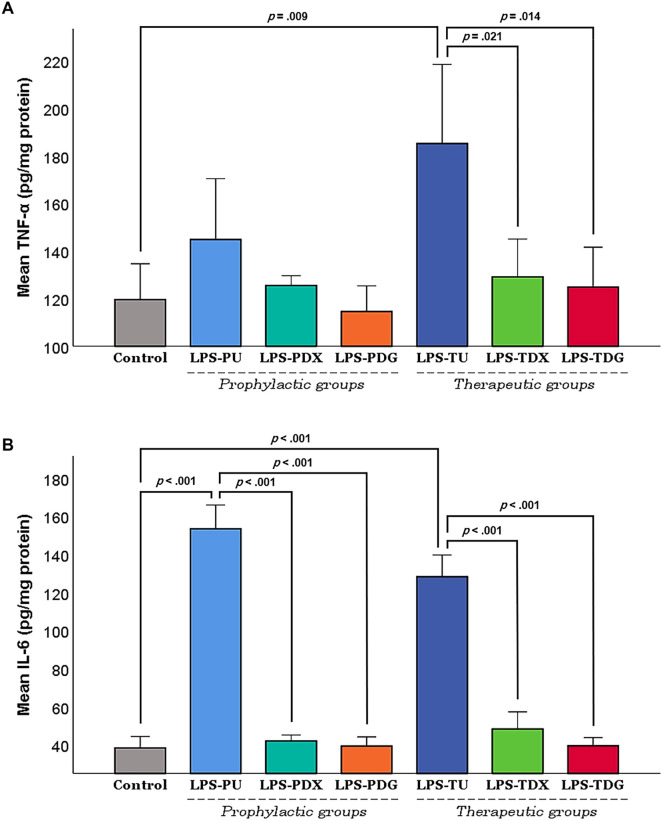
**Effect of digoxin on proinflammatory cytokines**. Results are presented as mean ± SEM. The levels of (A) TNF-α and (B) IL-6 were quantified in lung tissue homogenates at the study endpoint using appropriate ELISA assays. Values were normalized to total protein concentration to enhance accuracy and are reported as picograms per milligram of protein (pg/mg protein). Control mice (*n* ═ 10) were pooled equally from groups C1 and C2. The LPS groups comprised all mice administered intratracheal LPS (5 mg/kg) for the induction of ALI, divided into prophylactic and therapeutic subgroups. Prophylactic groups included: LPS-PU (untreated; *n* ═ 6), LPS-PDX (i.p. dexamethasone 5 mg/kg; *n* ═ 6), and LPS-PDG (i.p. digoxin 1 mg/kg; *n* ═ 6). Therapeutic groups included: LPS-TU (untreated; *n* ═ 6), LPS-TDX (i.p. dexamethasone 5 mg/kg; *n* ═ 6), and LPS-TDG (i.p. digoxin 1 mg/kg; *n* ═ 6). Brackets indicate Games-Howell *P* values (following Welch’s ANOVA). Abbreviations: ALI: Acute lung injury; ANOVA: Analysis of variance; ELISA: Enzyme-linked immunosorbent assay; i.p.: Intraperitoneal; LPS: Lipopolysaccharide; LPS-PDG: Lipopolysaccharide–prophylactic digoxin; LPS-PDX: Lipopolysaccharide–prophylactic dexamethasone; LPS-PU: Lipopolysaccharide–prophylactic untreated; LPS-TDG: Lipopolysaccharide–therapeutic digoxin; LPS-TDX: Lipopolysaccharide–therapeutic dexamethasone; LPS-TU: Lipopolysaccharide–therapeutic untreated; SEM: Standard error of the mean; TNF-α: Tumor necrosis factor-alpha.

As illustrated in Figure 3A, TNF-α levels in the prophylactic groups exhibited a minor increase in the LPS-PU group, with no statistically significant differences observed among all prophylactic groups (LPS-PU, LPS-PDX, and LPS-PDG). In contrast, the therapeutic groups demonstrated substantially elevated TNF-α levels in the untreated animals (LPS-TU) compared to baseline controls (*P ═* .009). Notably, treatment with either dexamethasone (LPS-TDX; *P ═* .021) or digoxin (LPS-TDG; *P ═* .014) resulted in a significant reduction of this inflammatory marker relative to the LPS-TU group.

Similarly, IL-6 levels exhibited significant variations across both the prophylactic and therapeutic groups (Figure 3B). The LPS-PU group showed a remarkable elevation in IL-6 levels, reaching up to fourfold compared to control mice (*P <* .001), whereas both prophylactic dexamethasone (LPS-PDX; *P <* .001) and digoxin (LPS-PDG; *P <* .001) groups demonstrated significantly suppressed levels relative to the LPS-PU group. Likewise, IL-6 levels were significantly elevated in the therapeutic untreated mice (LPS-TU; *P <* .001 vs control). Furthermore, both dexamethasone (LPS-TDX) and digoxin interventions (LPS-TDG) exhibited pronounced suppression of IL-6 levels relative to the LPS-TU group (*P <* .001).

### Effect of digoxin on MPO enzyme level and oxidative stress

**Figure 4.**
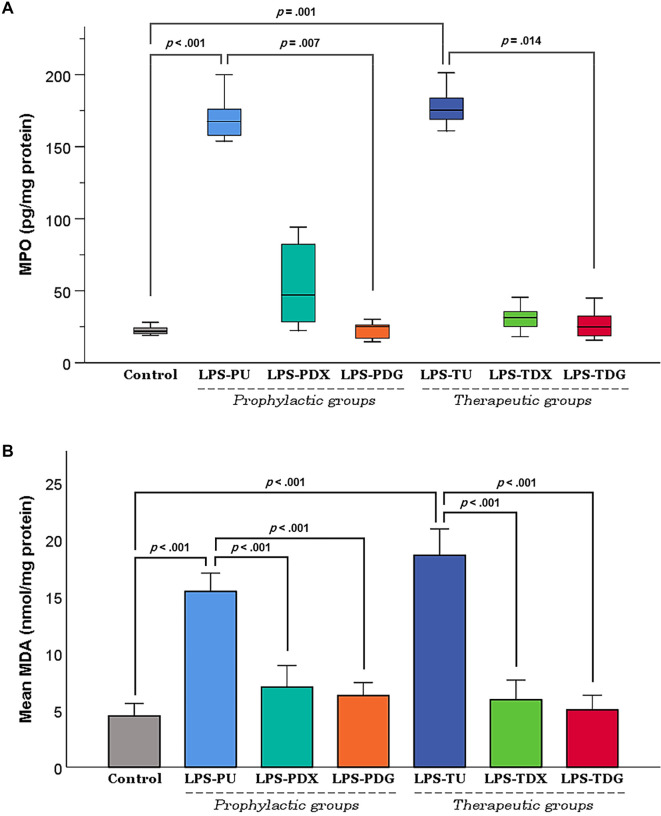
**Effect of digoxin on MPO and MDA in lung tissue homogenates at the study endpoint.** (A) MPO levels across experimental groups. (B) MDA levels across experimental groups. Data are presented as mean ± SEM; MPO and MDA values were standardized to total protein concentration and expressed per milligram of protein (/mg protein). Control mice (*n* ═ 10) were equally pooled from C1 and C2. The LPS groups included all mice receiving i.t. LPS (5 mg/kg) for ALI induction, subdivided into prophylactic and therapeutic subgroups. Prophylactic groups consisted of LPS-PU (untreated; *n* ═ 6), LPS-PDX (i.p. dexamethasone 5 mg/kg; *n* ═ 6), and LPS-PDG (i.p. digoxin 1 mg/kg; *n* ═ 6). Therapeutic groups included LPS-TU (untreated; *n* ═ 6), LPS-TDX (i.p. dexamethasone 5 mg/kg; *n* ═ 6), and LPS-TDG (i.p. digoxin 1 mg/kg; *n* ═ 6). Statistical analysis for MPO utilized Dunn-Bonferroni *P* values (Kruskal-Wallis), while MDA analysis employed Games-Howell p-values (Welch’s ANOVA). Abbreviations: ALI: Acute lung injury; ANOVA: Analysis of variance; i.p.: Intraperitoneal; i.t.: Intratracheal; LPS: Lipopolysaccharide; LPS-PDG: Lipopolysaccharide–prophylactic digoxin; LPS-PDX: Lipopolysaccharide–prophylactic dexamethasone; LPS-PU: Lipopolysaccharide–prophylactic untreated; LPS-TDG: Lipopolysaccharide–therapeutic digoxin; LPS-TDX: Lipopolysaccharide–therapeutic dexamethasone; LPS-TU: Lipopolysaccharide–therapeutic untreated; MDA: Malondialdehyde; MPO: Myeloperoxidase; SEM: Standard error of the mean.

Assessment of MPO enzyme levels (Figure 4A), a marker of neutrophil infiltration, revealed significant elevations in both diseased-untreated groups compared to controls (LPS-PU, *P <* .001; LPS-TU, *P ═* .001). Notably, the median values in these groups exhibited an approximately sevenfold elevation relative to baseline controls. In the prophylactic groups, administration of digoxin (LPS-PDG) resulted in a statistically significant reduction in MPO levels compared to the LPS-PU group (*P ═* .007), whereas LPS-PDX group did not achieve a meaningful decrease (*P ═* .59). In the therapeutic segment of the experiment, only digoxin (LPS-TDG) demonstrated significant reductions in MPO levels compared to the untreated group (*P ═* .014).

**Figure 5.**
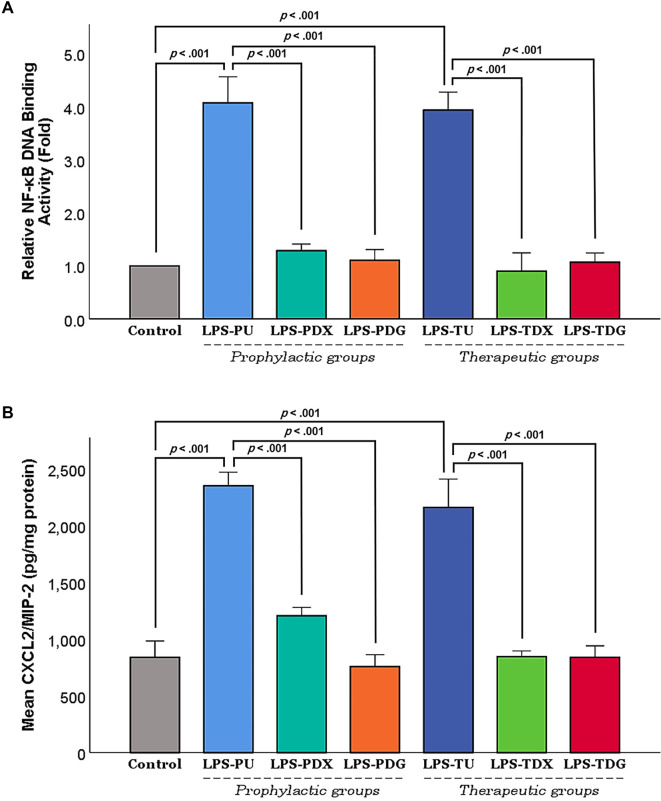
**Impact of digoxin on NF-κB activity and CXCL2/MIP-2 protein expression in lung tissue at the study endpoint.** (A) Relative NF-κB DNA-binding activity (fold change vs. control). (B) Lung tissue protein levels of macrophage inflammatory protein-2 (CXCL2/MIP-2) across experimental groups. Results are presented as mean ± SEM. Relative NF-κB DNA-binding activity is expressed as fold change compared to the control group. Levels of macrophage inflammatory protein-2 (CXCL2/MIP-2) in lung tissue homogenates were quantified, normalized to total protein content, and reported as picograms per milligram of protein (pg/mg protein). Control mice (*n* ═ 10) were equally pooled from groups C1 and C2. The LPS groups included all mice receiving i.t. LPS (5 mg/kg) for the induction of ALI, subdivided into prophylactic and therapeutic subgroups. Prophylactic groups consisted of LPS-PU (untreated; *n* ═ 6), LPS-PDX (i.p. dexamethasone 5 mg/kg; *n* ═ 6), and LPS-PDG (i.p. digoxin 1 mg/kg; *n* ═ 6). Therapeutic groups included LPS-TU (untreated; *n* ═ 6), LPS-TDX (i.p. dexamethasone 5 mg/kg; *n* ═ 6), and LPS-TDG (i.p. digoxin 1 mg/kg; *n* ═ 6). Brackets indicate Games-Howell *P*-values following Welch’s ANOVA. Abbreviations: ALI: Acute lung injury; ANOVA: Analysis of variance; CXCL2/MIP-2: C-X-C motif chemokine ligand 2/macrophage inflammatory protein-2; i.p.: Intraperitoneal; i.t.: Intratracheal; LPS: Lipopolysaccharide; LPS-PDG: Lipopolysaccharide–prophylactic digoxin; LPS-PDX: Lipopolysaccharide–prophylactic dexamethasone; LPS-PU: Lipopolysaccharide–prophylactic untreated; LPS-TDG: Lipopolysaccharide–therapeutic digoxin; LPS-TDX: Lipopolysaccharide–therapeutic dexamethasone; LPS-TU: Lipopolysaccharide–therapeutic untreated; NF-κB: Nuclear factor kappa B; SEM: Standard error of the mean.

As a principal end-product of lipid peroxidation, MDA serves as a reliable indicator of oxidative damage to cellular membranes and provides insight into the extent of free radical-mediated injury in the affected tissues. The MDA levels in this study exhibited trends parallel to other inflammatory markers (Figure 4B). Specifically, both the LPS-PU and LPS-TU groups demonstrated significant elevations in MDA concentrations compared to the control group (*P <* .001), indicating substantial oxidative stress in the lung tissues of diseased-untreated mice. In contrast, groups that received either dexamethasone (LPS-PDX and LPS-TDX) or digoxin (LPS-PDG and LPS-TDG) displayed lower MDA levels, with statistically significant differences (all *P <* .001) compared to the corresponding LPS-untreated groups (LPS-PU and LPS-TU). The alignment of MPO and MDA results further substantiates the reliability of each finding and corroborates the incidence of inflammatory responses along with oxidative stress in the LPS-untreated mice. The data also illustrate the critical coexistence of inflammation and oxidative stress in this pathological condition, alongside digoxin’s capacity to significantly mitigate both factors.

### Effect of digoxin on NF-κB activation, CXCL2/MIP-2 protein expression and the regulatory influence on HIF-1α levels

To evaluate the cellular mechanism of the intended intervention on the NF-κB pathway, nuclear NF-κB DNA binding activity was quantified across the various experimental mouse groups. As shown in Figure 5A, NF-κB binding activity was increased fourfold in both the LPS-PU and therapeutic (LPS-TU) untreated groups compared to the control group (*P <* .001). However, this elevated NF-κB activity observed in the prophylactic LPS-PU group was significantly inhibited by dexamethasone and digoxin, as demonstrated in the LPS-PDX and LPS-PDG groups, respectively (*P <* .001). Similarly, the therapeutic groups treated with either dexamethasone (LPS-TDX) or digoxin (LPS-TDG) exhibited significant suppression of NF-κB activity (*P <* .001), with levels aligning with those observed in the control group.

Furthermore, variations in NF-κB activity were reflected by corresponding changes in CXCL2/MIP-2 protein expression (Figure 5B). Specifically, CXCL2/MIP-2 protein levels increased significantly in the LPS-PU group compared to controls (*P <* .001). In contrast, both LPS-PDX and LPS-PDG groups revealed markedly lower levels of this protein relative to LPS-PU (*P <* .001). A similar pattern was observed in the therapeutic segment of the experiment: LPS-TU exhibited elevated CXCL2/MIP-2 levels compared to controls (*P <* .001), while therapeutic intervention (LPS-TDX/LPS-TDG) resulted in significant suppression relative to LPS-TU (*P <* .001).

**Figure 6.**
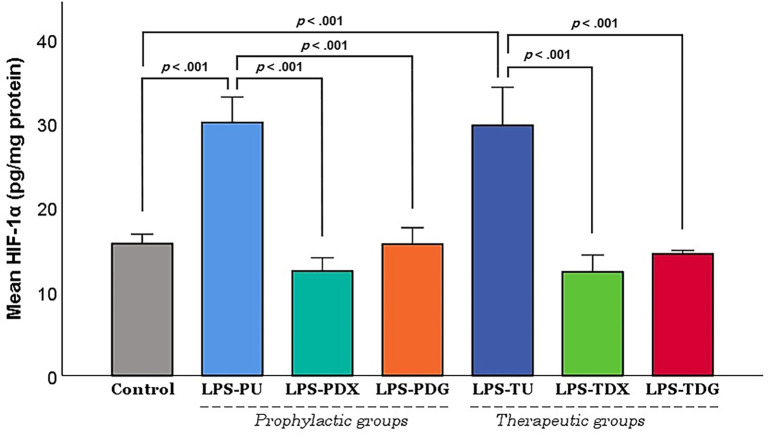
**The effect of digoxin on HIF-1α protein levels at study endpoint.** Results are presented as mean ± SEM. HIF-1α levels in lung tissue homogenates were quantified, normalized to total protein content, and expressed as picograms per milligram of protein (pg/mg protein). Control mice (*n* ═ 10) were pooled equally from groups C1 and C2. The LPS groups included all mice receiving i.t. lipopolysaccharide (LPS; 5 mg/kg) for ALI induction, subdivided into prophylactic and therapeutic subgroups. Prophylactic groups included LPS-PU (untreated; *n* ═ 6), LPS-PDX (i.p. dexamethasone 5 mg/kg; *n* ═ 6), and LPS-PDG (i.p. digoxin 1 mg/kg; *n* ═ 6). Therapeutic groups included LPS-TU (untreated; *n* ═ 6), LPS-TDX (i.p. dexamethasone 5 mg/kg; *n* ═ 6), and LPS-TDG (i.p. digoxin 1 mg/kg; *n* ═ 6). Statistical significance was determined using Games-Howell *P* values (Welch’s ANOVA). Abbreviations: ALI: Acute lung injury; ANOVA: Analysis of variance; HIF-1α: Hypoxia-inducible factor-1 alpha; i.p.: Intraperitoneal; i.t.: Intratracheal; LPS: Lipopolysaccharide; LPS-PDG: Lipopolysaccharide–prophylactic digoxin; LPS-PDX: Lipopolysaccharide–prophylactic dexamethasone; LPS-PU: Lipopolysaccharide–prophylactic untreated; LPS-TDG: Lipopolysaccharide–therapeutic digoxin; LPS-TDX: Lipopolysaccharide–therapeutic dexamethasone; LPS-TU: Lipopolysaccharide–therapeutic untreated; SEM: Standard error of the mean.

Additionally, the protein level of HIF-1α was evaluated to investigate its regulatory influence by NF-κB activity. Figure 6 shows that HIF-1α expression reached its peak in the LPS-PU prophylactic group, with values significantly exceeding those of the control (*P <* .001). In stark contrast, pre-treatment with dexamethasone (LPS-PDX) or digoxin (LPS-PDG) resulted in notably diminished HIF-1α concentrations compared to LPS-PU (*P <* .001). Likewise, HIF-1α concentrations peaked in the LPS-TU group, surpassing those observed in controls (*P <* .001). Furthermore, the administration of assigned interventions (LPS-TDX or LPS-TDG) led to a pronounced and significant decrease in HIF-1α levels relative to LPS-TU (*P <* .001). Thus, these data demonstrate that HIF-1α expression profiles closely correspond with NF-κB activation states in the current ALI model.

### Histopathological assessment of digoxin administration on lung tissue across experimental groups at study endpoint

**Figure 7.**
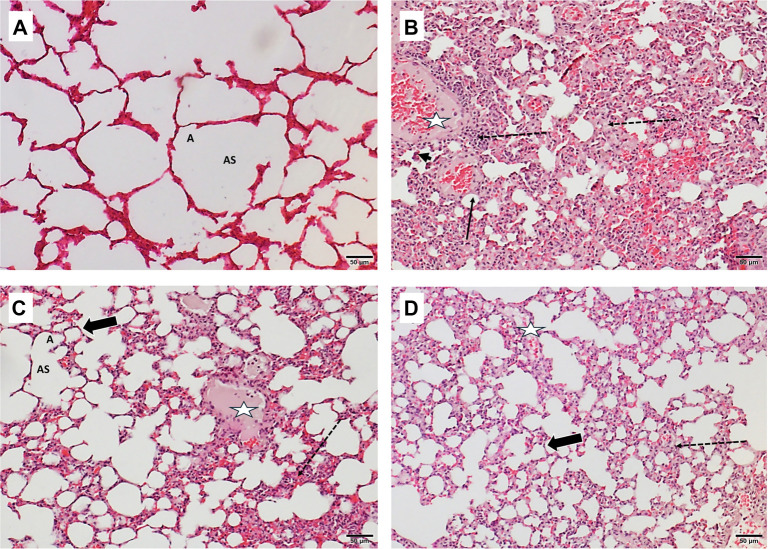
**Histopathological evaluation of lung tissues from prophylactic groups (H&E, 200×).** (A) Photomicrograph of lung section from the control group (C1) showing a normal structural pattern of the alveolar sacs (AS) and alveoli (A) with normal alveolar wall. (B) Photomicrograph of lung section from the prophylactically untreated group (LPS-PU) showing distorted lung structure with marked thickening of the alveolar walls, severe interstitial inflammatory infiltrates (dashed arrow), prominent hyaline membranes (arrow), edema and hemorrhage (star), as well as desquamated pneumocytes with reactive pneumocyte hyperplasia (black head arrow). (C) Photomicrograph of lung section from the prophylactic dexamethasone group (LPS-PDX) revealing localized areas of edema (star) and interstitial inflammatory infiltrates (dashed arrow), but several alveoli maintain normal thickness of the wall (thick arrow). (D) Photomicrograph of lung section from the prophylactic digoxin group (LPS-PDG) also illustrating localized edema (star) and interstitial inflammatory infiltrates (dashed arrow), with multiple alveoli showing preserved normal wall thickness (thick arrow). Mice involved in the LPS groups received intratracheal LPS (5 mg/kg) to induce ALI, including LPS-PU (untreated), LPS-PDX (prophylactic i.p. dexamethasone 5 mg/kg), and LPS-PDG (prophylactic i.p. digoxin 1 mg/kg). Abbreviations: A: Alveoli; ALI: Acute lung injury; AS: Alveolar sacs; H&E: Hematoxylin and eosin; i.p.: Intraperitoneal; LPS: Lipopolysaccharide; LPS-PDG: Lipopolysaccharide–prophylactic digoxin; LPS-PDX: Lipopolysaccharide–prophylactic dexamethasone; LPS-PU: Lipopolysaccharide–prophylactic untreated.

In the prophylactic groups (Figure 7), evaluation of lung photomicrographs from the control group revealed a normal structural pattern of the alveolar sacs and alveoli, with the alveolar walls appearing intact and unremarkable. In contrast, the LPS-PU group exhibited significantly distorted lung architecture, characterized by pronounced thickening of the alveolar walls, extensive interstitial inflammatory cell infiltration, and notable development of hyaline membranes. Additionally, edema and hemorrhagic changes were present, alongside desquamation of pneumocytes. In comparison, these pathological manifestations were less pronounced in the LPS-PDX and LPS-PDG groups (Table 2), where localized areas of edema were observed, and both the extent of interstitial inflammatory infiltrates and the presence of hyaline membranes were less conspicuous. Unexpectedly, LPS-PDX exhibited more airspace edema than LPS-PDG, while capillary congestion was less evident in LPS-PDG. A subtle reduction in inflammatory cell accumulation was noted in LPS-PDX relative to LPS-PDG. However, the presence of normal wall thickness in some alveoli, combined with reduced hemorrhage and accumulation of inflammatory cells indicates a protective effect in both groups compared to LPS-PU-induced damage.

**Table 2:** Histopathological scoring of lung injury per mouse across different experimental groups at study conclusion

**Groups**			**Mouse #**	**Airspace edema**	**Thickening of alveolar septa and type II pneumocyte hyperplasia**	**Hemorrhage**	**Congestion of capillaries**	**Prominent hyaline membrane**	**Accumulation of Inflammatory cells**
**Control**			M-1	–	–	–	–	–	–
			M-2	–	–	–	–	–	–
**LPS groups** **^a^**	*Prophylactic groups*	**LPS-PU**	M-1	1	4	2	4	2	4
			M-2	1	4	4	4	3	4
		**LPS-PDX**	M-1	3	4	1	4	2	2
			M-2	1	3	–	4	2	2
			M-3	2	3	–	3	2	1
			M-4	2	3	–	4	1	2
		**LPS-PDG**	M-1	1	3	–	2	2	2
			M-2	2	3	–	4	2	3
			M-3	–	3	–	4	1	3
			M-4	1	2	–	3	2	3
	*Therapeutic groups*	**LPS-TU**	M-1	1	4	3	4	3	4
			M-2	1	4	3	3	3	4
		**LPS-TDX**	M-1	1	3	3	4	–	1
			M-2	1	3	2	3	–	1
			M-3	2	3	4	4	–	1
			M-4	–	4	2	4	1	2
		**LPS-TDG**	M-1	–	2	2	3	–	2
			M-2	–	2	3	4	–	3
			M-3	1	3	3	3	1	1
			M-4	–	1	2	4	–	2

**Notes**: The histopathological scoring system was adapted from Frank et al. [39], incorporating modifications that prioritized parameters most relevant to our model. The grading criteria were also simplified to improve reproducibility and facilitate interpretation. Control mice included one each from groups C1 and C2 (n = 2). The LPS groups consisted of all mice receiving i.t. LPS (5 mg/kg) for ALI induction, subdivided into prophylactic and therapeutic subgroups. The prophylactic groups included LPS-PU (untreated), LPS-PDX (i.p. dexamethasone 5 mg/kg), and LPS-PDG (i.p. digoxin 1 mg/kg). The therapeutic groups comprised LPS-TU (untreated), LPS-TDX (i.p. dexamethasone 5 mg/kg), and LPS-TDG (i.p. digoxin 1 mg/kg). Scores indicate the extent of parenchymal involvement: absent (–); 1 = 1–24%; 2 = 25–49%; 3 = 50–74%; 4 = 75–100%. Abbreviations: ALI: Acute lung injury; i.p.: Intraperitoneal; i.t.: Intratracheal; LPS: Lipopolysaccharide; LPS-PDG: Lipopolysaccharide–prophylactic digoxin; LPS-PDX: Lipopolysaccharide–prophylactic dexamethasone; LPS-PU: Lipopolysaccharide–prophylactic untreated; LPS-TDG: Lipopolysaccharide–therapeutic digoxin; LPS-TDX: Lipopolysaccharide–therapeutic dexamethasone; LPS-TU: Lipopolysaccharide–therapeutic untreated.

**Figure 8.**
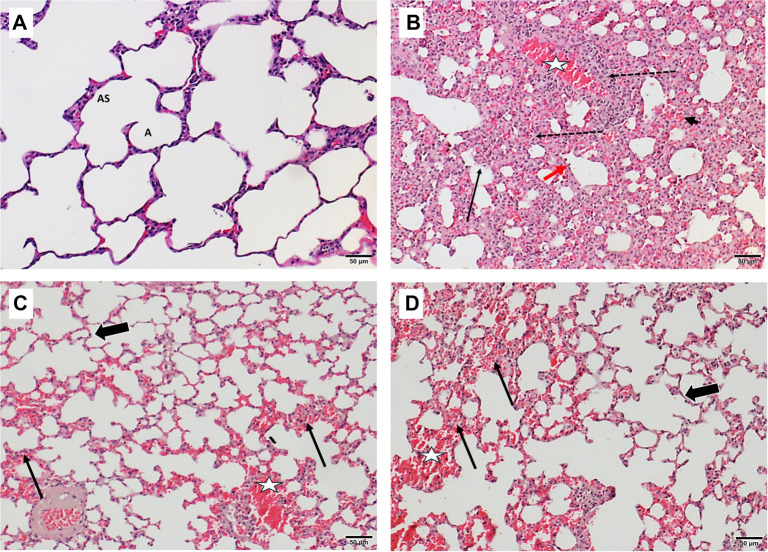
**Histopathological assessment of lung tissue from therapeutic groups (H&E, 200×).** (A) Photomicrographs of lung sections from the control group (C2) show well-defined alveolar sacs (AS) and alveoli (A) with intact, thin alveolar walls, indicative of normal histological architecture. (B) The LPS-TU exhibited a distorted lung structure with marked thickening of the alveolar walls, severe interstitial inflammatory infiltrates (dashed arrow), prominent hyaline membranes (arrow), hemorrhage (star), marked capillary congestion (black head arrow) and reactive pneumocyte hyperplasia (red head arrow). (C) Group LPS-TDX reveals localized areas of hemorrhage (star), sporadic thickening of the alveolar walls with capillary congestion (arrows) but numerous alveoli exhibiting normal thickness of the wall (thick arrow). (D) Group LPS-TDG also exhibits localized hemorrhage (star), alveolar wall thickening with capillary congestion (arrows), yet many alveoli retain normal wall thickness (thick arrow). Mice involved in the LPS groups received intratracheal LPS (5 mg/kg) to induce ALI, including LPS-TU (untreated), LPS-TDX (therapeutic i.p. dexamethasone 5 mg/kg), and LPS-TDG (therapeutic i.p. digoxin 1 mg/kg). Abbreviations: A: Alveoli; ALI: Acute lung injury; AS: Alveolar sacs; H&E: Hematoxylin and eosin; i.p.: Intraperitoneal; LPS: Lipopolysaccharide; LPS-TDG: Lipopolysaccharide–therapeutic digoxin; LPS-TDX: Lipopolysaccharide–therapeutic dexamethasone; LPS-TU: Lipopolysaccharide–therapeutic untreated.

**Figure 9.**
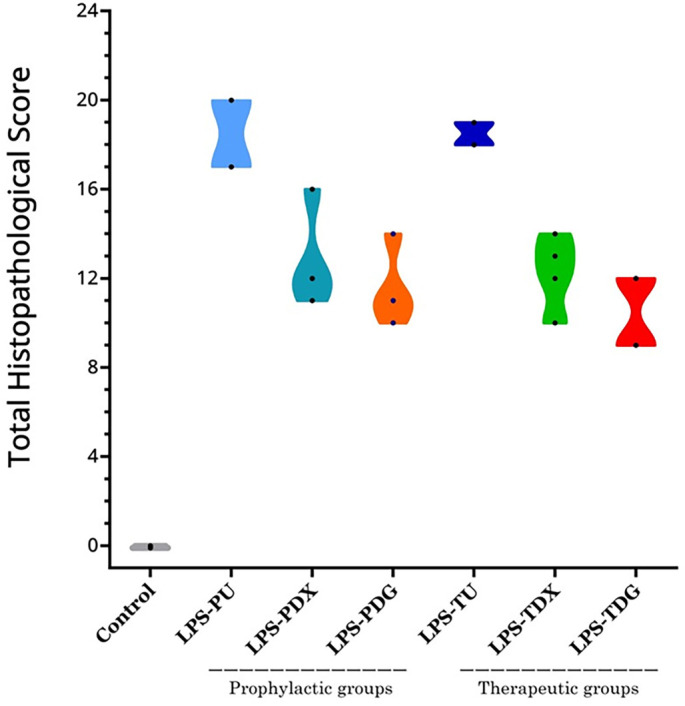
**Total histopathological lung injury scores by group.** The absence of individual pathological features was scored as 0 in the total histopathological scores calculated per mouse. Control mice (*n* ═ 2; one from C1 and one from C2) exhibited no discernible histopathological features, resulting in total scores of 0. The LPS groups comprised all mice that received i.t. LPS (5 mg/kg) for the induction of ALI, categorized into prophylactic and therapeutic subgroups. Prophylactic groups included LPS-PU (untreated; *n* ═ 2), LPS-PDX (i.p. dexamethasone 5 mg/kg; *n* ═ 4), and LPS-PDG (i.p. digoxin 1 mg/kg; *n* ═ 4). Therapeutic groups consisted of LPS-TU (untreated; *n* ═ 2), LPS-TDX (i.p. dexamethasone 5 mg/kg; *n* ═ 4), and LPS-TDG (i.p. digoxin 1 mg/kg; *n* ═ 4). Abbreviations: ALI: Acute lung injury; i.p.: Intraperitoneal; i.t.: Intratracheal; LPS: Lipopolysaccharide; LPS-PDG: Lipopolysaccharide–prophylactic digoxin; LPS-PDX: Lipopolysaccharide–prophylactic dexamethasone; LPS-PU: Lipopolysaccharide–prophylactic untreated; LPS-TDG: Lipopolysaccharide–therapeutic digoxin; LPS-TDX: Lipopolysaccharide–therapeutic dexamethasone; LPS-TU: Lipopolysaccharide–therapeutic untreated.

Conversely, the therapeutic groups demonstrated a high degree of concordance in their histopathological findings (Figure 8). The LPS-TU group displayed significant architectural disruption of the lung parenchyma, marked by pronounced thickening of the alveolar walls, severe interstitial inflammatory cell infiltration, and prominent hyaline membranes. Additional pathological findings included areas of hemorrhage, marked capillary congestion, and evidence of reactive pneumocyte hyperplasia. In contrast, the LPS-TDX and LPS-TDG groups exhibited comparable hemorrhage and capillary congestion but showed reduced alveolar wall thickening and fewer infiltrating inflammatory cells. Further inter-group assessment revealed that the LPS-TDG group exhibited reduced airspace edema and better preservation of the alveolar wall, while the LPS-TDX group demonstrated a slightly lower accumulation of inflammatory cells. Notably, a substantial proportion of alveoli retained their architecture, and inflammatory cell infiltration was reduced in both LPS-TDX and LPS-TDG groups. Combined with lower total histopathological scores (Figure 9), these findings indicate partial preservation of lung parenchyma by both interventions.

## Discussion

ARDS represents a significant health concern arising from various etiologies, with pneumonia being one of the most prevalent causes. Key pathological features include the overexpression of inflammatory mediators, increased oxidative damage, disruption of pulmonary vascular permeability, pronounced neutrophil infiltration, and compromised gas exchange. The consequences of these syndromes can be severe, resulting in increased patient morbidity, mortality, and substantial strain on the healthcare system. The existing insufficiency of pharmacologic treatments underscores the need for ongoing research aimed at improving management strategies for these conditions [43–45]. Accordingly, the present study aimed to investigate the preclinical prophylactic and therapeutic potential of digoxin in a mouse model of ALI.

Intratracheal LPS instillation in mice serves as a valuable ALI model, replicating robust neutrophil infiltration, elevated proinflammatory mediators, and capillary-alveolar membrane disruption, which are key features of human ARDS pathology [26, 27, 46]. In this study, intratracheal administration of LPS induced pronounced ALI, as evidenced by several key findings. Both prophylactic and therapeutic LPS-untreated groups (LPS-PU and LPS-TU) exhibited markedly reduced weight gain following LPS exposure, reflecting the systemic impact of the ALI model. The lung wet-to-dry ratio, particularly in the LPS-TU group, was significantly increased, indicating pronounced pulmonary edema characteristic of ALI. Biochemical analysis of lung homogenates confirmed ALI pathology, revealing elevated inflammatory cytokines (IL-6 and therapeutic-phase TNF-α), higher neutrophil infiltration markers, and increased oxidative stress. Histopathological examination corroborated these findings, showing alveolar wall thickening, edema, inflammation, and hyaline membrane formation. Collectively, these results underscore the effectiveness of the LPS-induced ALI model in replicating critical disease aspects and provide a robust foundation for evaluating digoxin as a potential therapeutic intervention.

In the current investigation, LPS exposure significantly impaired the mice’s ability to achieve weight gain comparable to that of normal mice. The observed weight changes align with prior reports, driven by systemic inflammation that suppresses appetite and alters metabolism [47]. Notably, digoxin appeared to influence body weight changes, as both prophylactic and therapeutic digoxin-treated mice (LPS-PDG, LPS-TDG) exhibited lower median weight changes than LPS-untreated groups, with significantly lower variation compared to dexamethasone-treated mice (see Figure S1). The further reduction in weight gain among digoxin-treated groups corroborates earlier findings on its mitigation of diet-induced obesity. Digoxin dose-dependently inhibits weight gain, adiposity, hypercholesterolemia, steatosis, and liver injury by blocking RAR-related orphan receptor gamma t (RORγt)-mediated IL-17A production [29]. Remarkably, this pathway is also implicated in ALI pathogenesis and has been proposed as a potential therapeutic strategy [48].

The lung wet-to-dry weight ratio reliably quantifies pulmonary edema and injury by measuring tissue water content. Its validity is supported by widespread use and correlations with arterial oxygenation and alveolar-capillary permeability [49]. In this study, both prophylactic and therapeutic untreated groups (LPS-PU, LPS-TU) exhibited increased lung wet-to-dry ratios, although statistical significance was detected only in the therapeutic group (LPS-TU). This suggests that the 18-hour prophylactic interval was insufficient for full pulmonary edema development at the current LPS dose, unlike the 72-hour therapeutic period. This aligns with prior studies that reported insignificant wet-to-dry ratios under similar conditions [50]. Importantly, the LPS challenge in the therapeutic arm of the study (LPS-TU) precipitated remarkable pulmonary edema, consistent with a broader body of existing literature [51–53]. In this therapeutic phase, only digoxin significantly reduced edema. The LPS-TDG group’s results closely resembled those of normal control mice, suggesting markedly enhanced edema resolution. Combined with histopathological scoring, these findings suggest that digoxin may possess a distinct anti-edema mechanism compared to dexamethasone under these conditions.

Inflammation is central to both ALI and ARDS, featuring the massive release of proinflammatory cytokines that induce alveolar damage, vascular leakage, and impaired gas exchange. TNF-α, particularly prominent, plays a key role in activating apoptosis, inhibiting pathogen replication, and recruiting immune cells [54]. Serum TNF-α levels increase progressively across respiratory conditions, being highest in ARDS patients, intermediate in pneumonia, and lowest in healthy controls [55]. Remarkably, the current TNF-α levels in the LPS-PU group showed only a modest, non-significant increase. Previous evidence has demonstrated that TNF-α release is markedly time-dependent, with rapid peaks occurring within hours post-LPS followed by a decline, reflecting complex regulatory dynamics [56, 57]. Additionally, rabbit lung studies confirm that TNF-α release remains largely localized to administration sites [58]. When LPS is delivered to the alveolar space, TNF-α is primarily released there, with minimal vascular spillover, supporting the compartmentalized response observed in the current prophylactic experimental phase.

However, in cases of unresolved inflammation or injury, TNF-α concentrations remain significantly elevated for days. In a mouse model of acute pancreatitis, serum TNF-α levels increased by 24 h, reached statistical significance at 48 h, and peaked at 72 h. These elevations paralleled increases in pulmonary intercellular adhesion molecule-1 (ICAM-1) and vascular cell adhesion molecule-1 (VCAM-1), both critical to the inflammatory response [59]. Furthermore, in spinal cord injury patients, serum TNF-α initially decreased at 4 h (nadir at 9 h), rose significantly at 72 h, declined during week 2, and then increased again at 4 weeks post-injury [60]. Collectively, the evidence indicates that sustained inflammatory stimuli or injury may lead to renewed or extended TNF-α expression beyond the initial phase of the inflammatory response. The current investigation supports this concept by demonstrating a significant elevation of TNF-α levels in the therapeutic untreated group (LPS-TU), indicative of a sustained inflammatory response. In contrast, treatment with digoxin resulted in a substantial reduction in TNF-α levels, demonstrating efficacy comparable to that of dexamethasone.

Furthermore, IL-6 serves as an adjunctive marker to TNF-α, which is widely employed to enhance the assessment of inflammatory status. Unlike TNF-α, IL-6 exhibits superior sensitivity and a significant correlation with C-reactive protein [61–63]. Current IL-6 levels align with other parameters, validating the model and supporting digoxin’s anti-inflammatory effect. Macrophages are primary producers of IL-6, and their activation during ALI plays a critical role in eliciting a robust inflammatory response. IL-6 amplifies inflammation, stimulates T and B cells, and promotes the differentiation of naïve T cells into T helper 17 (Th17) cells [64], which produce IL-17, thereby linking this discussion to the previously suggested mechanism of digoxin in facilitating weight loss. Additionally, targeting IL-6 with tocilizumab (initially approved for rheumatoid arthritis) has been shown to reduce COVID-19 mortality and ventilation requirements in critically ill patients [65]. Consequently, the observed inhibitory effect of digoxin on IL-6 in this study further reinforces its promising potential for pulmonary protection.

Equally important, MPO levels demonstrated significant increases in the LPS-untreated groups (LPS-PU and LPS-TU). Digoxin exhibited suppressive effects in both prophylactic and therapeutic experiments. MPO critically mediates acute lung inflammation through neutrophil extravasation and recruitment, as evidenced by studies involving MPO-deficient mice following LPS challenge [66]. MPO serves as a reliable marker of inflammation, exhibiting sensitivity comparable to IL-6 and superior to histopathology for detecting acute-phase responses [67]. Recent evidence has highlighted MPO as a key inflammatory marker in COVID-19, with levels elevated in acute cases correlating with disease severity. Notably, *in vitro* MPO inhibition reduced endothelial glycocalyx shedding, suggesting a potential therapeutic approach to limit vascular damage [68].

In the present study, digoxin administration resulted in a significant decrease in MPO levels, reflecting a marked attenuation of pulmonary neutrophilia, corroborated by histopathological analysis. MPO resides in neutrophil azurophilic granules, fusing with phagosomes to combat pathogens upon activation. However, degranulation releases MPO extracellularly, leading to tissue damage and inflammation [69]. LPS binds to Toll-like receptor 4 (TLR4) on neutrophils, triggering myeloid differentiation primary response 88 (MyD88)-dependent NF-κB activation. This induces the expression of pro-inflammatory mediators and extends neutrophil survival [70]. Consequently, enhanced inflammation amplifies MPO release and neutrophil activation via sustained cytokine signaling. MPO further perpetuates this cycle through autocrine and paracrine mechanisms [69]. The current findings indicate that digoxin effectively suppresses MPO, TNF-α (during the therapeutic phase), and IL-6 production, breaking this cycle of persistent inflammation.

In the context of oxidative stress, neutrophil-derived MPO exacerbates oxidative damage to pulmonary epithelium by converting hydrogen peroxide and chloride into hypochlorous acid (HOCl). MPO/HOCl dysregulate elastase, generate reactive oxygen species, and induce pyroptosis, collectively destroying pulmonary connective tissue [71]. Furthermore, studies using MPO-deficient mice have demonstrated reduced disease severity and neutrophilic oxidative damage in pulmonary inflammatory conditions without impairing bacterial clearance [72]. In the present study, administration of digoxin resulted in a significant reduction in oxidative stress, evidenced by decreased levels of both MPO and MDA. The concurrent inhibition of these biomarkers further demonstrates that digoxin effectively mitigates oxidative stress induced by ALI.

Importantly, digoxin in the current investigation also exhibited inhibitory effects on NF-κB activation at the nuclear level, consistent with findings reported in previous studies [17–20]. In fact, digoxin’s inhibition of NF-κB signaling is a recognized anti-cancer mechanism involving the downregulation of NF-κB-associated genes that promote cell growth and survival [73]. Additionally, in this study, digoxin markedly suppressed the NF-κB–dependent CXCL2/MIP-2 expression, a principal chemokine driving neutrophil recruitment and subsequent lung inflammation during pulmonary infection [74–76]. Therefore, the downstream inhibition of the NF-κB signaling axis may serve as a subcellular mechanism underlying the observed effects of digoxin. NF-κB inhibition by digoxin reduces CXCL2/MIP-2 expression, attenuating neutrophil influx, MPO overproduction, inflammation, and oxidative stress. NF-κB also regulates the transcription of TNF-α and IL-6 in immune cells through nuclear translocation and genomic binding [77]. Thus, digoxin’s inhibition of NF-κB signaling may represent a pivotal junction that broadens the mechanistic explanation to include the observed suppression of TNF-α and IL-6 in this study.

Additionally, digoxin suppressed HIF-1α, a key transcription factor that promotes hypoxic adaptation but may also exacerbate inflammation [23]. Interestingly, HIF-1α may facilitate SARS-CoV-2 infection and promote COVID-19 inflammatory responses [78]. Moreover, inhibition of HIF-1α has been proposed as a therapeutic strategy for ALI, as its overactivation amplifies lung inflammation and alveolar epithelial apoptosis [79, 80]. In fact, our finding of digoxin-mediated HIF-1α inhibition in ALI extends prior demonstrations of this effect in tumor [81] and chronic obstructive pulmonary disease (COPD) models [82]. Accordingly, digoxin’s effect on HIF-1α expression complements and extends the previously discussed protective mechanisms against LPS-induced ALI.

Histopathological evaluation provided further confirmation of digoxin’s pulmonary protective potential, with greater resolution of airspace edema than dexamethasone in the therapeutic experiment. It also demonstrated comparable preservation of alveolar wall thickness, marked reduction of inflammatory infiltration, and diminished hyaline membrane formation.

Literature confirms the expression of Na^+^/K^+^-ATPase (digoxin’s molecular target) in lung tissue, where it facilitates alveolar fluid clearance. Type I pneumocytes, which cover most of the alveolar surface, express α1 and α2 isoforms [83]. However, cardiac glycosides like digoxin exhibit preferential binding to specific Na^+^/K^+^-ATPase isoforms (α1 vs. α2), with affinity varying by compound and potassium concentration [84]. In mice, a 50% reduction of either isoform alone spared alveolar fluid clearance, but dual reduction caused significant impairment, suggesting that inhibition of both isoforms is necessary [85]. Furthermore, alveolar fluid clearance involves multifaceted regulation beyond Na^+^/K^+^-ATPase, including catecholamine-dependent and independent pathways, cytokines, aquaporins, chloride transport, and alveolar-capillary barrier integrity, providing compensatory mechanisms that maintain fluid homeostasis [86, 87]. Moreover, large clinical trials [88] and systematic reviews/meta-analyses of observational studies [89] found no significant increase in isolated pulmonary edema incidence among digoxin-treated patients. The current findings demonstrating digoxin’s efficacy against edema not only reinforce this perspective but also provide evidence supporting its paradoxical positive effects, challenging established theoretical assumptions.

Our extensive literature review indicates that no prior research has investigated the potential of digoxin against LPS-induced ALI. We acknowledge the narrow therapeutic index and toxicity risks associated with digoxin; however, these concerns are beyond the preclinical exploratory scope of the present study and therefore were not the focus of our investigation. The dosage employed in the present study was justified by previous investigations that demonstrated anti-inflammatory effects [29, 30]. Although effective in this murine ALI model, this dose represents a species-specific concentration that exceeds human-relevant exposure levels, consistent with rodents’ relative resistance to digitalis toxicity [90, 91]. Additionally, while the intratracheal LPS model effectively recapitulates the early inflammatory phase of ARDS, it does not fully capture the multifactorial pathophysiology, prolonged resolution phase, or mechanical ventilation component characteristic of clinical ARDS. Also, the histopathological scoring was performed unblinded, with predefined semi-quantitative criteria applied to each feature assessed. Furthermore, serum digoxin levels and cardiotoxicity were not assessed in this study. Future research could address this gap through systematic dose-ranging evaluations with corresponding safety profiles. Moreover, the potential additive or synergistic effects of combining digoxin with dexamethasone in this condition warrant further investigation. Additional elucidation of the subcellular mechanisms underlying NF-κB downstream inhibition and its crosstalk with other signaling pathways remains a critical area of interest.

## Conclusion

The present study establishes digoxin’s immunomodulatory efficacy in a mouse model of LPS-induced lung injury. Digoxin revealed significant benefits in addressing key pathological features of ALI, including improved resolution of pulmonary edema, suppression of pro-inflammatory cytokines (IL-6 and therapeutic-phase TNF-α), reduced neutrophil infiltration, and decreased oxidative stress. These protective effects of digoxin were further validated by histopathological findings. The current outcomes stem, at least in part, from digoxin’s inhibition of NF-κB signaling and downstream inflammatory proteins, along with reduced expression of HIF-1α. The multifaceted therapeutic actions of digoxin on key pathological features of ALI provide a scientific rationale for future investigations to validate these findings alongside dose optimization and toxicity mitigation strategies.

## Supplemental data

Supplemental data are available at the following link: https://www.bjbms.org/ojs/index.php/bjbms/article/view/13786/4132.

## Data Availability

The data supporting the results of this study can be obtained from the corresponding author upon reasonable request.
